# Human *Rickettsia sibirica mongolitimonae* Infection, Spain

**DOI:** 10.3201/eid1403.070987

**Published:** 2008-03

**Authors:** Koldo Aguirrebengoa, Aránzazu Portillo, Sonia Santibáñez, Juan J. Marín, Miguel Montejo, José A. Oteo

**Affiliations:** * Hospital de Cruces, Baracaldo, Spain; † Hospital San Pedro–Centro de Investigación Biomédica de La Rioja (CIBIR), Logroño, Spain

**Keywords:** Rickettsia sibirica mongolitimonae, Spain, tick-borne disease, letter

## Abstract

Human *Rickettsia sibirica mongolitimonae* Infection, Spain

**To the Editor:** *Rickettsia sibirica mongolitimonae* has been recently reported as a subspecies of *R. sibirica* (*1*). The first evidence of *R. sibirica mongolitimonae* pathogenicity in humans was documented in France in 1996 (*2*). Since then, 11 more cases in France, Algeria, South Africa, Greece, and Portugal have been reported (*3*–*6*). Because the main clinical manifestations include lymphangitis, the acronym LAR (lymphangitis-associated rickettsiosis) has been proposed (*3*). We report a case from Spain that confirms the broad distribution of this agent in southern Europe.

A 41-year-old man was admitted on June 19, 2007, to the Hospital de Cruces (Baracaldo, Spain) with fever (39°C), malaise for a week, sweating, lumbar and knee pain, disseminated myalgias, and headache. He reported that 20 days before admission he had removed an engorged tick from his right leg while working as a topographer in the Balmaseda Mountains, 30 km from Bilbao. He had also removed several ticks from his body 4 days before the onset of symptoms. Physical examination did not demonstrate relevant findings. There was no inoculation eschar at the tick-bite sites. Rash, lymphadenopathies, and lymphangitis were not observed.

Chest radiograph did not show consolidation or other abnormality. Initial laboratory examination, on June 21, 2007, showed a leukocyte count 5.2 × 10^3^/μL, hemoglobin 14.1 g/dL, platelet count 190,000/μL, erythrocyte sedimentation speed 9 mm/h, urea 38 mg/dL, creatinine 0.9 mg/dL, aspartate aminotransferase 229 IU/L, alanine aminotransferase 170 IU/L, alkaline phosphatase 158 IU/L, gamma-glutamyl-transpeptidase 111 IU/L, total bilirubin 1.3 mg/dL, and C-reactive protein 4.3 mg/dL. Because the patient had been bitten by a tick, acute-phase serum and EDTA-treated blood samples were sent to the Special Pathogens Laboratory (Área de Enfermedades Infecciosas – Hospital San Pedro from La Rioja), where a presumptive diagnosis of rickettsiosis was made. On June 22, 2007, treatment with doxycycline was begun (100 mg/day for 12 days), and his condition rapidly improved.

The early-phase serum yielded low immunoglobulin (Ig) G titer (<64) against *Rickettsia conorii* and *Anaplasma phagocytophilum* antigens, and results of ELISA and Western blotting for Lyme borreliosis were negative. A convalescent-phase serum sample collected 7 weeks later did not contain IgG antibodies against spotted fever group *Rickettsia* species when *R. conorii* antigen was used.

DNA was extracted from the early whole-blood specimen by using QIAamp DNA Blood minikit (QIAGEN, Hilden, Germany) according to the manufacturer’s instructions. This DNA extract was used as template in nested PCR assays targeting the spotted fever group rickettsial *ompB* (420 bp) and *gltA* (337 bp) genes (*7*). Quality control included both positive (with *R. conorii* Malish #7 grown in Vero cells) and negative controls that were extracted and PCR amplified in parallel with the specimens. Negative controls consisted of sterile water instead of template DNA. Amplification products of the expected size were obtained. The sequences of these amplicons allowed the identification of *R. sibirica mongolitimonae* with 99.5% and 100% similarity for *ompB* and *gltA*, respectively (GenBank accession nos. DQ097083 and DQ097081).

To our knowledge, *Rickettsia* species have never been detected in ticks or human specimens in Spain. The host ticks of this rickettsia are likely *Hyalomma* species, which are more prevalent in southern Spain. In our region in northern Spain, *Hyalomma marginatum* represented 8% of ticks that fed on humans during 2001–2005, although an increase in this number was recorded last year (data not shown).

In our patient, *Rickettsia*’s pathogenic role was demonstrated by PCR, a technique that has previously enabled us to identify other arthropod-borne *Rickettsia* species (*8*,*9*). This case suggests that *R. sibirica mongolitimonae* infection should be considered in the differential diagnosis of rickettsiosis and tick-bite febrile patients in Spain and confirms the distribution of this rickettsia in southern Europe. According to the literature (*3*), some patients in whom *R. sibirica mongolitimonae* infection is diagnosed have >1 eschar, which raises the suspicion that some cases of Mediterranean spotted fever with multiple eschars reported in Spain could be caused by this rickettsial species. More studies about the vectors of this bacteria are needed because studies of *Hyalomma* and *Rhipicephalus* ticks (the suspected hosts) conducted in our area have not demonstrated the presence of this *Rickettsia* species.
